# Complete Genome Sequence of Pigmentation-Negative *Yersinia pestis* Strain Cadman

**DOI:** 10.1128/genomeA.01207-16

**Published:** 2016-10-27

**Authors:** Sean Lovett, Kitty Chase, Galina Koroleva, Gustavo Palacios, David Rozak, Jason T. Ladner

**Affiliations:** a Center for Genome Sciences, U.S. Army Medical Research Institute of Infectious Diseases, Fort Detrick, Frederick, Maryland, USA; b Diagnostic Systems Division, U.S. Army Medical Research Institute of Infectious Diseases, Fort Detrick, Frederick, Maryland, USA

## Abstract

Here, we report the genome sequence of *Yersinia pestis* strain Cadman, an attenuated strain lacking the *pgm* locus. *Y. pestis* is the causative agent of plague and generally must be worked with under biosafety level 3 (BSL-3) conditions. However, strains lacking the *pgm* locus are considered safe to work with under BSL-2 conditions.

## GENOME ANNOUNCEMENT

*Yersinia pestis* is the causative agent of plague. Due to its high potential for use in biological terrorism, the CDC has classified it a category A pathogen, which must be handled within a biosafety level 3 laboratory.

Here, we report the complete sequence of the pigmentation-negative (*pgm*−) Cadman strain of *Y. pestis*. This strain was isolated in 1965 from cerebrospinal fluid obtained from a 2.5-year-old boy (1). It is unknown whether the original isolate was *pgm*− or whether this mutation occurred during laboratory passage. However, naturally occurring *pgm−* isolates have been associated with disease in humans and rodents (2). The virulence of *pgm−* strains of *Y. pestis* is attenuated (3) and therefore they can be manipulated under less restrictive laboratory conditions.

Chromosomal DNA was prepared using the Qiagen EZ1 Advanced XL system with the EZ1 DNA tissue kit according to manufacturer’s instructions. The sequencing library was prepared using the SMRTbell template prep kit (Pacific Biosciences, Menlo Park, CA) following manufacturer’s protocol. The resulting SMRTbell template was size selected on BluePippin system (Sage Science, Beverly, MA) using 0.75% dye-free agarose cassette with 4 to 10 kb Hi-Pass protocol and low cut set at 4 kb. Size selected template was cleaned and concentrated with AMPure PB beads. The P4 polymerase was used with the C2 sequencing kit to generate 240-minute movies.

Raw sequencing reads were filtered for a minimum subread length of 1,000 bp, minimum polymerase read length of 5,000 bp, and minimum quality of 0.80. The filtered data set contained 101,466 reads with a mean subread length of 8,409 bp.

Reads were assembled into four contigs using HGAP3 v2.3 (4). These contigs correspond to the chromosome and plasmids pCD1, pPCP1, and pMT1. Each contig was checked for redundant sequence at the ends using Gepard v1.3 (5). The plasmids were determined to be complete circular molecules, so redundant sequences at the ends were trimmed to one copy and a new breakpoint was chosen for their linear representations. A large (~50 kb) repeat on the chromosome was not able to be resolved, so the chromosome was not modified. Reads were then realigned to the trimmed and shifted draft assembly for correction using Quiver (4).

Annotation with NCBI’s Prokaryotic Genome Annotation Pipeline v3.3 (6) identified 4,238 coding regions, 19 ribosomal RNAs, 68 tRNAs, 12 noncoding RNAs (ncRNAs), and two clustered regularly interspaced short palindromic repeat (CRISPR) arrays. Gene content was compared to *Y. pestis* CO92 (NC_003131, NC_003132, NC_003134, NC_003143). Nucleotide sequences of annotated genes were clustered at 90% identity using USearch (7). Genes without assigned orthologs were used as queries in a BLAST search against both genomes. Genes with ≥90% nucleotide identity to a region of the CO92 genome were considered to be present, even if unannotated. All annotated genes outside of the *pgm* locus in CO92 were present in Cadman, although some exhibit alterations that may result in loss of function. No genes present in Cadman were absent in CO92. Absence of the *pgm* locus was confirmed by aligning all reads to the *pgm* + CO92 using BWA-mem v0.7.12 (8). No significant alignments to sequence unique to the *pgm* locus were observed.

### Accession number(s).

Complete sequences for all molecules have been deposited at GenBank under the accession numbers CP016273 to CP016276.
